# Comparison of blood loss between intra-articular and intra-venous administration of tranexamic acid in primary total knee arthroplasty

**DOI:** 10.1051/sicotj/2020017

**Published:** 2020-06-17

**Authors:** Muhammad Suhail Amin, Muhammad Khurram Habib, Aziz Ur Rehman

**Affiliations:** 1 Professor Orthopedic, CMH Hospital Rawalpindi and CMH Lahore Orthopedic ward, CMH Hospital Rawalpindi Pakistan; 2 Assistant Professor Orthopedics, DHQ Hospital Faisalabad, Clinical Fellow, CMH Lahore, DHQ Hospital Faisalabad Orthopedics ward Pakistan; 3 Clinical fellow CMH Hospital Rawalpindi and CMH Lahore Pakistan

**Keywords:** Primary Total Knee Arthroplasty, Tranexamic Acid, Intra-articular, Blood Loss

## Abstract

*Objective*: To compare the blood loss between intra-articular and intra-venous administration of tranexamic acid (TXA) in patients undergoing primary total knee arthroplasty. *Design of study*: It was a randomized controlled trial. *Study duration and settings*: This study was carried out at the Orthopedic Departments of Combined Military Hospital Lahore and Rawalpindi from Jan 2016 to March 2018. *Methodology*: Patients of both the genders were involved this study who had age in the rage of 40–80 years undergoing primary unilateral total knee arthroplasty for degenerative conditions like osteoarthritis and rheumatoid arthritis. These patients were randomly divided into two treatment groups. Patients in IA group received intra-articular tranexamic acid while those in IV group received intravenous tranexamic acid. From all the patients, a written signed consent was taken. *Findings*: Females were predominant with male-to-female ratio of 1:3.7. The mean age of the patients was 67.3 ± 8.2 years while the mean BMI was 30.9 ± 2.9 Kg/m^2^. Majority (*n* = 191, 95.5%) of the patients had osteoarthritis while remaining 9 (4.5%) patients had rheumatoid arthritis. There was no statistically significant difference between intra-articular and intra-venous administration of tranexamic acid in terms of mean post-operative hemoglobin (9.93 ± 1.14 vs. 9.87 ± 1.26 g/dL; *p*-value = 0.724), mean post-operative hematocrit (34.8 ± 1.66 vs. 34.73 ± 1.27%; *p*-value = 0.594), and mean fall in hemoglobin (2.27 ± 0.34 vs. 2.25 ± 0.30 g/dL; *p*-value = 0.587) and hematocrit (2.34 ± 0.94 vs. 2.46 ± 0.28%; *p*-value = 0.216). *Conclusion*: Intra-articular administration of tranexamic acid was found to be as effective and safe as intra-venous administration in reducing blood loss in primary total knee arthroplasty. Due to convenience, the use of intra-articular administration of tranexamic acid after primary TKA may be considered in future practice.

## Introduction

Despite that advancements in fixation and design techniques of implants have substantially increased functional results and survival rates of total knee arthroplasty (TKA), during the procedure loss of blood is a major concern that is peri-operatively estimated to be 850–1600 mL [1]. During TKA, Patient’s recovery may be affected negatively by blood loss. Allogenic blood transfusion may be needed on the basis of amount of blood lost during the procedure [2]. Moreover, high post-operative infections are also associated with this procedure [3], measures which can reduce operative loss of blood and subsequent necessitating blood transfusion have always been adored and a hot focus of medical research [2].

From reducing operative blood loss, promising results have been given by tranexamic acid (TXA) among the various techniques used, till now [4]. Being synthetic amino acid, tranexamic acid functions by competitive inhibition of plasminogen conversion into plasmin, thus promoting clot stabilization [4, 5]. Followed by total knee arthroplasty, tranexamic acid has got identity as effective antifibrinolytic helpful in decreasing blood loss and transfusion risks. Moreover, it has not been associated with increased thromboembolic complications [6–28].

Surgeons may choose to administer tranexamic acid intravenously, topically, or orally [6]. Though, in total knee arthroplasty settings involving tranexamic acid, majority of the studies have focused on the intravenous route [6–21], topical spraying of TXA has been proved to be equally efficacious while being safer as far as post-operative problems like DVT are concerned [6–25]. The existing evidence on the efficacy of this topical application contained controversy where some studies claimed no difference between IV and IA routes in terms of blood loss [6–20], other showed significant difference and advocated intra-articular application [21–24] while still others reported intravenous route to be better and associated with significantly lower operative blood loss [25–28]. A major limitation to these studies was limited sample size of less than 30–50 cases. Moreover, this study has also been necessitated by non-availability of locally published such material.

## Material and methods

The present study was RCT (randomized controlled trial) carried out at the Orthopedic Departments of Combined Military Hospital Lahore and Rawalpindi from Jan 2016 to March 2018. Each group was comprised of 100 cases in this study having 200 cases sample size calculated with 95% significance level and 80% power of test considering expected mean post-operative hemoglobin to be 11.0 ± 0.67 g/dL in patients with intravenous and 10.6 ± 1.26 g/dL in patients with intra-articular tranexamic acid [28]. In this study, patients had age between 40 and 80 years belonging to both the genders suffering from advanced degenerative disease of knee planned for unilateral total knee replacement (TKR). However, patients with complex and revision complex knee arthroplasty, single stage bilateral total knee replacement, and unicompartmental knee arthroplasty were excluded from the study. By random allocation of these patients, two equal-sized treatment groups were made. Patients in IA group received 2 g of TXA in 20 mL solution injected by syringe into the joint after the prosthesis was inserted and cemented and the entire operative field was thoroughly rinsed and dried, no drain was placed and wound closed in layers before skin closure. Patients in IV group received intravenous dose of 1 g TXA administered 5–10 min before the pneumatic tourniquet was inflated [29].

This procedure was performed by a senior surgeon through standardized techniques. Under a high thigh tourniquet, a medial parapatellar and middle line incision approach was used during all the operative procedures. In all the cases, patella was resurfaced and tibial and femoral components were cemented. In all patients, PCL-sacrificing posterior stabilized knee prosthesis was used. They included: Vanguard^®^ (Biomet), Genesis II^®^ (Smith & Nephew) and PFC-Sigma (DePuy, Johnson & Johnson) NexGen^®^ (Zimmer, Ltd., Swindon, UK). The procedures were performed either under spinal anesthesia or general anesthesia always combined with an epidural. Before the closure of the wound, the tourniquet was deflated and hemostasis secured. No drain was placed in both groups. After layered closure and intra-articular injection in IA group, a compression bandage with the knee in extension was given, which stayed for about six hours post-operatively. Routine DVT (deep vein thrombosis) prophylaxis comprised of LMWH that was started 12 h pre-operatively and was continued for 30 days after the surgery.

Outcome variables were mean post-operative hemoglobin, mean post-operative hematocrit, and mean fall in hemoglobin and hematocrit all measured 24 h after surgery. Numerical variables like age, BMI, hematocrit, pre-operative and post-operative hemoglobin, and fall in hematocrit and hemoglobin have been described as mean ± SD. By taking *p*-value ≤ 0.05 as statistically significant, for comparing mean of these various variables in the study group, independent sample test *t*-test was applied. Categorical variable like gender has been described as frequency and percentage. In order to minimize biasness, standard operative technique was used and a single surgeon performed all these procedures.

## Results

Mean age of the patients was 67.3 ± 8.2 years with age ranging 48–80 years. The study group had 43 (21.5%) male patients and 157 (78.5%) female patients with a male-to-female ratio of 1:3.7. The BMI of the patients ranged from 24.0 Kg/m^2^ to 34.9 Kg/m^2^ with a mean of 30.9 ± 2.9 Kg/m^2^. Majority (*n* = 191, 95.5%) of the patients had osteoarthritis while remaining 9 (4.5%) patients had rheumatoid arthritis. The two study groups were homogenous in terms of mean age (*p*-value = 0.979), mean BMI (*p*-value = 0.439), mean pre-operative hemoglobin (*p*-value = 0.606), mean pre-operative hematocrit (*p*-value = 0.953) and gender (*p*-value = 0.863) and etiologic (*p*-value = 0.733) groups distribution (Table 1). In terms of mean post-operative hematocrit (*p*-value = 0.594), post-operative hemoglobin (*p*-value = 0.724) and mean fall in hematocrit (*p*-value = 0.216) and hemoglobin (*p*-value = 0.587) no statistically significant difference was noted between IA and IV TXA. These findings have been summarized in Table 2.

**Table 1:** Demographic features of study groups.

Characteristics	IA *n* = 100	IV *n* = 100	*P* value
Age (years)	67.3 ± 8.3	67.3 ± 8.1	0.979
Gender			
Male	22 (22.9%)	21 (21.0%)	0.863
Female	78 (78.0%)	79 (79.0%)	
BMI (Kg/m^2^)	31.0 ± 2.7	30.7 ± 3.1	0.439
Cause			
Osteoarthritis	95 (95.8%)	96 (94.1%)	0.733
Rheumatoid arthritis	5 (4.2%)	4 (5.9%)	

Chi-square test, Independent sample *t*-test, no significant difference was observed (*p* > 0.05).

**Table 2:** Comparison of various outcome measures.

Characteristics	IA *n* = 100	IV *n* = 100	*P* value
Pre-operative Data			
Pre-operative hemoglobin (g/dL)	12.20 ± 1.12	12.12 ± 1.20	0.606
Pre-operative hematocrit (%)	37.18 ± 1.22	37.19 ± 1.20	0.953
Post-operative data			
Post-operative hemoglobin (g/dL)	9.93 ± 1.14	9.87 ± 1.26	0.724
Post-operative hematocrit (%)	34.8 ± 1.66	34.73 ± 1.27	0.594
Fall in hemoglobin (g/dL)	2.27 ± 0.34	2.25 ± 0.30	0.587
Fall in hematocrit (%)	2.34 ± 0.94	2.46 ± 0.28	0.216

Independent sample *t*-test, observed difference was statistically insignificant.

## Discussion

Different measures to reduce operative blood loss have been employed in orthopedic surgery to reduce the need for transfusion and hasten the patient’s recovery in the post-operative period [2]. A frequently employed such technique is transfusion of autologous blood which also reduces the risk of infection, but it is expensive and not every center has the facility [2, 3]. Another method for control of the perioperative blood loss is the application of anti-fibrinolytic agents including aprotinin, epsilon-aminocaproic acid, and tranexamic acid (TXA) [4]. Among them, TXA has gained the maximum attention which can be given intravenous or intra-articular during the surgery [4]. The available evidence on the selection of more appropriate route of administration of TXA bears controversy. Moreover, present study was also necessitated by non-availability of locally published such material.

In the present study, we observed that the mean age of patients was 67.3 ± 8.2 years. Khan et al. [30] reported similar mean age of 64 ± 6.3 years in patients undergoing total knee replacement at Ghurki Trust Teaching Hospital Lahore while Obaid-ur-Rahman et al. [31] reported it to be 64 ± 8.4 years in such patients at Combined Military Hospital, Rawalpindi. A similar mean age of 67 ± 7.96 years has been reported by Maniar et al. [25] in Indian such patients while Pinsornsak et al. [16] reported it to be 67.63 ± 7.96 years in Thailand. Keyhani et al. [14] and Sarzaeem et al. [27] reported comparable mean age of 67 ± 11.9 years and 67.5 ± 7.6 years in Iran while Seo et al. [23] reported it to be 67.5 ± 6.6 years in Korea.

We observed a female predominance in patients undergoing TKR with a male-to-female ratio of 1:3.7. A similar female predominance among TKR patients has been reported by Chen et al. [11] in Singapore who reported a male-to-female ratio of 1:4. Digas et al. [22] and Drosos et al. [12] reported similar female predominance with male-to-female ratio of 1:3.3 and 1:4 respectively in Greece. Pinsornsak et al. [16] reported much higher female predominance with male-to-female ratio of 1:5 while Aggarwal et al. [21] reported a male predominance (m:f, 1.9:1) in India.

In the present study, we observed insignificant difference between intra-articular and intra-venous tranexamic acid in terms of mean post-operative hemoglobin and hematocrit and mean fall in hemoglobin and hematocrit. Our results are comparable to those of Digas et al. [22] who also reported similar insignificant difference between IA and IV TXA in terms of mean fall in hemoglobin after TKR (2.26 ± 0.99 vs. 2.24 ± 0.93 g/dL; *p*-value = 0.72). Similar results have also been published by Soni et al. [18] (2.21 ± 0.64 vs. 2.42 ± 0.86 g/dL; *p*-value = 0.38), Tzatzairis et al. [19] (2.95 ± 1.33 vs. 3.2 ± 1.29 g/dL; *p*-value = 0.551), Seo et al. [23] (1.8 ± 0.8 vs. 1.6 ± 0.8 g/dL; *p*-value > 0.05), Patel et al. [9] (3.42 ± 1.07 vs. 3.06 ± 1.02 g/dL; *p*-value = 0.108), Pinsornsak et al. [16] (1.85 ± 0.95 vs. 1.87 ± 1.37 g/dL; *p*-value = 0.840) and Sarzaeem et al. [27] (3.9 ± 1.1 vs. 2.6 ± 0.9 g/dL; *p*-value > 0.05). A similar insignificant difference in the mean post-operative hematocrit between IA and IV TXA has been reported by Aguilera et al. [10] (34.69 ± 3.42 vs. 34.05 ± 4.53%; *p*-value = 0.073). No significant difference in terms of mean post-operative hematocrit with IA and IV TXA in patients undergoing TKR has also been reported by Tzatzairis et al. [19] (32.44 ± 3.33 vs. 30.92 ± 3.11%; *p*-value = 0.112), Drosos et al. [12] (33.19 ± 2.97 vs. 32.41 ± 5.20%; *p*-value = 0.197) and Pinsornsak et al. [16] (31.0 ± 2.7 vs. 31.8 ± 3.4%; *p*-value = 0.352).

In local population, this type of study has been conducted for the first time and it supports international research already published on this topic. Fairly larger sample size of 200 cases and randomization of study groups to minimize bias which makes the results of the present study more reliable are strengths of this study. From evidence, intra-articular tranexamic acid preference over intravenous route as intra-articular route has been advocated. It is as efficacious as intravenous route, yet it avoids the inconvenience of IV administration and possibly avoids the complication of intravenous administration of TXA like deep vein thrombosis, myocardial infarction, pulmonary embolism, and cerebrovascular events [32].

A biggest limitation associated with this study was non comparing the effects of intra-venous and intra-articular tranexamic acid on coagulation profile as it can be expected that intra-articular tranexamic acid could not have as much deleterious effects as expected from intravenous administration which would further favor the use of intra-articular route. For future researches, this study is recommended strongly.

## Conclusion

In primary total knee arthroplasty, to reduce blood loss, intra-articular administration of tranexamic acid was found to be as effective and safe as intra-venous administration. Due to convenience, the use of intra-articular administration of tranexamic acid after primary TKA may be considered in future practice.
